# Facial expression monitoring system for predicting patient’s sudden movement during radiotherapy using deep learning

**DOI:** 10.1002/acm2.12945

**Published:** 2020-06-09

**Authors:** Kwang Hyeon Kim, Kyeongyun Park, Haksoo Kim, Byungdu Jo, Sang Hee Ahn, Chankyu Kim, Myeongsoo Kim, Tae Ho Kim, Se Byeong Lee, Dongho Shin, Young Kyung Lim, Jong Hwi Jeong

**Affiliations:** ^1^ Proton Therapy Center National Cancer Center Goyang Korea

**Keywords:** convolutional neural network, facial expression recognition, patient monitoring system, predicting body movement, Radiotherapy

## Abstract

**Purpose:**

Imaging, breath‐holding/gating, and fixation devices have been developed to minimize setup errors so that the prescribed dose can be exactly delivered to the target volume in radiotherapy. Despite these efforts, additional patient monitoring devices have been installed in the treatment room to view patients’ whole‐body movement. We developed a facial expression recognition system using deep learning with a convolutional neural network (CNN) to predict patients’ advanced movement, enhancing the stability of the radiation treatment by giving warning signs to radiation therapists.

**Materials and methods:**

Convolutional neural network model and extended Cohn‐Kanade datasets with 447 facial expressions of source images for training were used. Additionally, a user interface that can be used in the treatment control room was developed to monitor real‐time patient's facial expression in the treatment room, and the entire system was constructed by installing a camera in the treatment room. To predict the possibility of patients' sudden movement, we categorized facial expressions into two groups: (a) uncomfortable expressions and (b) comfortable expressions. We assumed that the warning sign about the sudden movement was given when the uncomfortable expression was recognized.

**Results:**

We have constructed the facial expression monitoring system, and the training and test accuracy were 100% and 85.6%, respectively. In 10 patients, their emotions were recognized based on their comfortable and uncomfortable expressions with 100% detection rate. The detected various emotions were represented by a heatmap and motion prediction accuracy was analyzed for each patient.

**Conclusion:**

We developed a system that monitors the patient's facial expressions and predicts patient's advanced movement during the treatment. It was confirmed that our patient monitoring system can be complementarily used with the existing monitoring system. This system will help in maintaining the initial setup and improving the accuracy of radiotherapy for the patients using deep learning in radiotherapy.

## 1 INTRODUCTION

The goal of radiotherapy is to irradiate the tumor target with the prescribed dose and minimize the exposure of radiation to the organ‐at‐risk (OAR) adjacent to the tumor on the radiation beam irradiation path.^1^ In the process of establishing a radiation treatment plan, when defining a tumor target, a planning target volume (PTV) usually includes uncertainty margin considering the irradiation errors and setup errors. Additionally, the margin change causes severe error, that is, the radiation dose can be irradiated to the normal tissue adjacent to the target volume. Moreover, uncertainty through system error and random error may arise in the treatment planning stage and the actual treatment process of the patient.^2^, ^3^ Therefore, images such as cone‐beam computed tomography and megavoltage computed tomography are obtained periodically at the beginning of and during the treatments, and the respiratory gated radiotherapy is used to minimize errors caused by internal organ motion.^4^, ^5^ Moreover, methods for improving the accuracy of radiation treatment using various immobilizers have been used to minimize body movement during radiation treatment.^6^, ^7^ Additionally, the patients are monitored in a radiation therapy control room using an installed video imaging device inside the treatment room. However, it is difficult to prevent patients from changing their postures in units of several millimeters to several centimeters and sudden movements. Therefore, development of a system is needed to inform the radiation treatment technologist regarding the emotional state of a patient by providing an audiovisual alarm. Certainly, various tools for surface imaging are available, and they can detect patient’s real‐time movement. However, these tools are expensive and hard to install in every treatment room.

Recognition of facial expressions can be applied to a wide range of research areas, such as diagnosing mental disorders and detecting human social/physiological interactions. Affective expressions can be shown in emotions, and these emotions are expressed in various ways by faces, gestures, postures, voices, and actions. It can also affect physiological parameters. Therefore, understanding emotional expression is important when performing diagnostic and therapeutic procedures.^8^, ^9^, ^10^ Fantoni and Gerbino^11^ conducted a cognitive psychological experiment where subjects could either comfortably or uncomfortably touch a visually induced object to identify facial emotions at each time. This indicates that there is an association between the patient’s facial expression and the comfortable/uncomfortable posture of the body. Since the emotional identification of the facial expression is associated with physical movement, the patient’s physical condition is inferred by extracting the uncomfortable emotion, which in turn enables patient monitoring. Shakya et al. studied the prediction of human behaviors through the recognition of facial expressions.^12^ In their study, artificial intelligence (AI) algorithm was used for facial expression recognition, and appropriate actions were predicted from various series of emotions.

There are several face recognition research projects using facial image databases (Table 1). In the early 1970s, Ekman P. introduced specific guidelines for research about face‐emotion connection and its judgment and analysis for psychology, anthropology, ethology, sociology, and biology. Recently, facial recognition is widely used in our practical life such as high‐security alert.^12^ The main purpose of the 2000s research was to obtain a vast amount of facial expression databases. However, the databases were finally applied to predict human behavior change through facial expression recognition to warn.^13^, ^14^, ^15^, ^16^

**Table 1:** Face recognition researches using facial images.

Authors	Goal	Result	Algorithm	Facial database	Year
Shakya et al.^12^	Human behavior prediction	Emotion analysis and its appropriate behavior prediction	Machine vision (PCA)	Extended Cohn‐Kanade dataset (CK+)	2016
Benitez‐Quiroz et al.^13^	Automatic annotation of facial expressions in the wild	Emotional annotation for a million facial expression images	Machine vision (Shape feature)	EmotioNet	2016
Pantic et al.^14^	Web‐based database for facial expression analysis	automatic analysis of facial expressions	Facial action coding system (FACS)	MMI facial expression dataset	2005
Ekman^15^	Research for emotion in the human face	Face‐emotion connection and its judgment and analysis for psychology, anthropology, ethology, sociology, and biology	N/A	Sampling persons	1972

In the radiation treatment process, before the treatment, the patient is advised by the radiation therapist not to move his/her body while the radiation treatment is in progress. However, in reality, one cannot directly control the movement of the patient undergoing treatment apart from the auditory notice using a microphone in a control room. Therefore, an AI algorithm using facial expression recognition of the patient in the abovementioned case can be used. By using alarms for patients who are uncomfortable in real time, a system that provides visual and auditory alerts and alarms to radiation therapist on the emotional state of the patient can prevent the occurrences of negative clinical outcomes as a result of patient’s movements by giving warning signs on the possibility about the sudden movement of the patient.

In this study, we aimed to develop a system that recognizes a patient’s facial expression, detects the patient's discomfort feelings using deep learning, alerts radiation therapy technologists on the possibility of sudden movement due to the patient’s discomfort in advance, and confirms the possibility of its utilization in the treatment of patients.

## 2 MATERIALS AND METHODS

In this study, it is necessary to construct a system that recognizes facial expressions of patients, detects uncomfortable feelings, and gives alarms to predict sudden patient movements. That is, in order to recognize the facial expression of the patient, a model capable of recognizing the facial expression was built using the convolutional neural network (CNN) which is a representative algorithm used for image classification among artificial intelligence algorithms. And a vision system was used to assign the actual facial expressions to the artificial intelligence algorithm. In addition, a CNN model is needed training and validation images of various expressions. And extended Cohn‐Kanade datasets (CK+) were used to optimize our system capacity and effectively recognize patient expression.^12^, ^13^, ^14^, ^15^, ^16^, ^17^

### 2.A System configuration

**Fig. 1:**
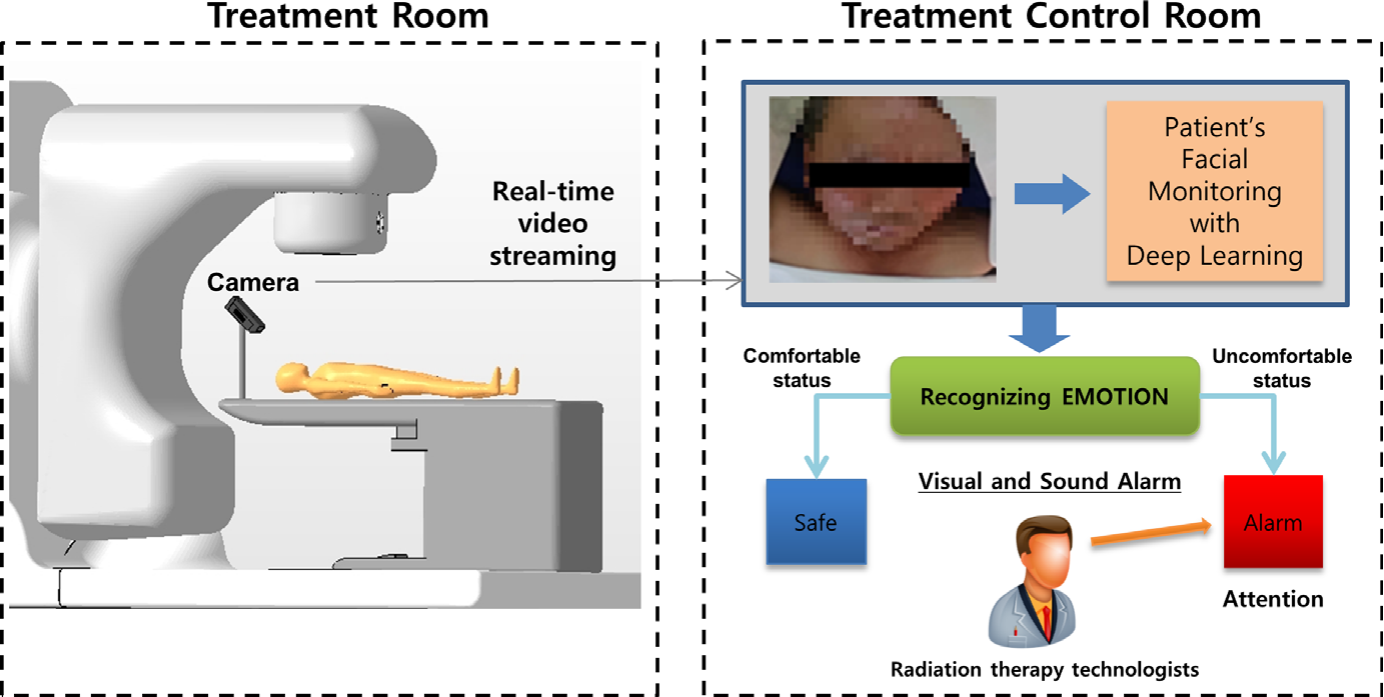
A facial expression monitoring system for patients in a radiation treatment room.

A vision system was established in the treatment room and treatment control room for facial expression monitoring. First, a charge‐coupled device camera (30 frames/s) for monitoring the facial expression of a patient in the treatment room was installed, and a personal computer (PC) with software for recognizing facial expressions of a patient in the treatment control room was installed (Fig. 1).

**Fig. 2:**
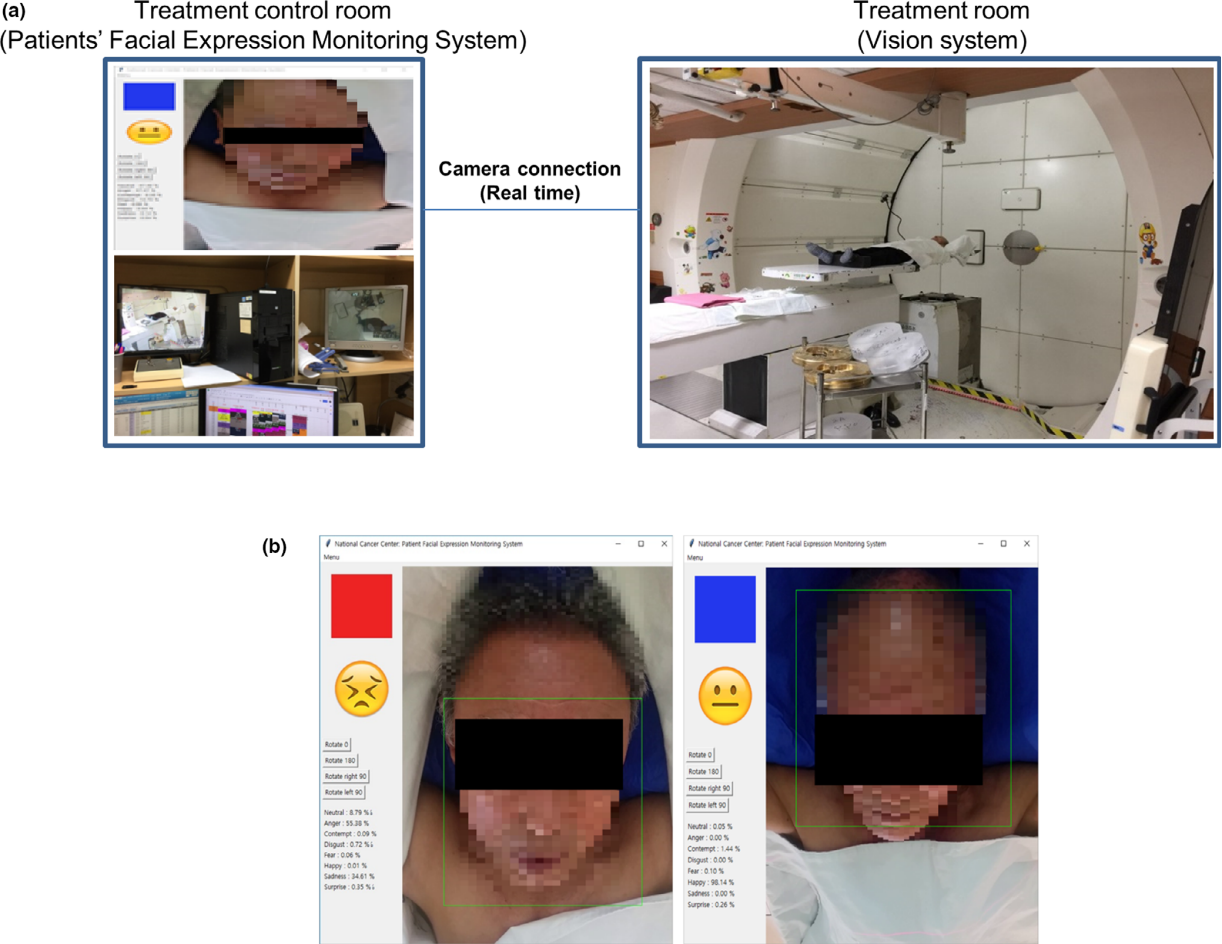
System implementation and a user interface.

The system constructed in the treatment control room and treatment room is shown in Fig. 2. A camera was installed in the treatment room to monitor the face of the patient, and a PC equipped with an machine learning algorithm was installed in the treatment control room [Fig. 2(a)]. The user interface is designed to display blue and red warning signs when it recognizes a comfortable or uncomfortable condition caused by facial expression [Fig. 2(b)].

### 2.B Convolutional neural network

**Fig. 3:**
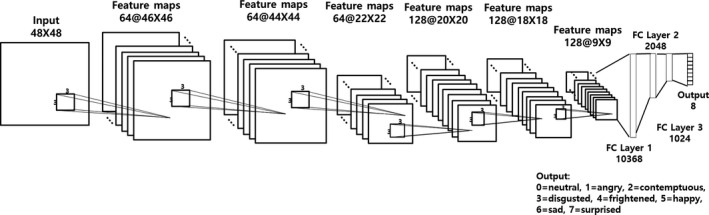
A developed convolutional neural network architecture.

For the machine learning algorithm, we used a CNN to classify patients’ facial expressions with images (Fig. 3). The CNN algorithm is widely used in computer vision processing and is one of the machine learning algorithms used for image‐based pattern recognition.^18^, ^19^, ^20^ Convolutional neural network is modeled on a computer in a way that detects the features of the target in the biological nervous system and comprehensively judges and recognizes them. CNN consists of a number of interconnected neurons, and out of those neurons, it is composed of three convolutional layers, pooling layers, and three fully connected layers, which are connected for the collective learning from the input, optimizing the final output.^12^, ^21^, ^22^, ^23^ The convolutional layer is an area that finds the features of the input signal. In the case of facial images, the convolutional layer detects the boundary and colors of the object. The deeper the layers, the more comprehensive and complex the facial expressions detected are. The pooling layer performs downsampling along the spatial dimension of a given input to reduce the amount of signal transmitted. This reduces the amount of computation required and enhances the detected features. The fully connected layer collects the extracted features to determine or classify the final input object. The $y_{i}^{\left(l\right)}$ as i^th^ feature map in convolutional layer l comprises $m_{1}^{\left(l-1\right)}$ feature maps from former layer for the $B_{i}^{\left(l\right)}$ , a bias matrix with the filter, $k_{i,j}^{\left(l\right)}$ is computed as

(1) $$y_{i}^{\left(l\right)}=B_{i}^{\left(l\right)}+\sum_{j=1}^{m_{1}^{\left(l-1\right)}}k_{i,j}^{\left(l\right)}*y_{j}^{\left(l-1\right)}$$

To construct the algorithm, we used Python environment (Anaconda) and Keras Deep Learning Library. The constructed CNN is shown in Fig. 3. The classifier was constructed with eight expressions through network from the 48 × 48 images input. To implement CNN that can receive and process video in real time, a network was configured in which there are six featured maps with convolution, rectified linear unit, and the pooling layers as one bundle and three fully connected layers that determine the extracted features. The network finally classifies the emotions in eight categories.

**Fig. 4:**
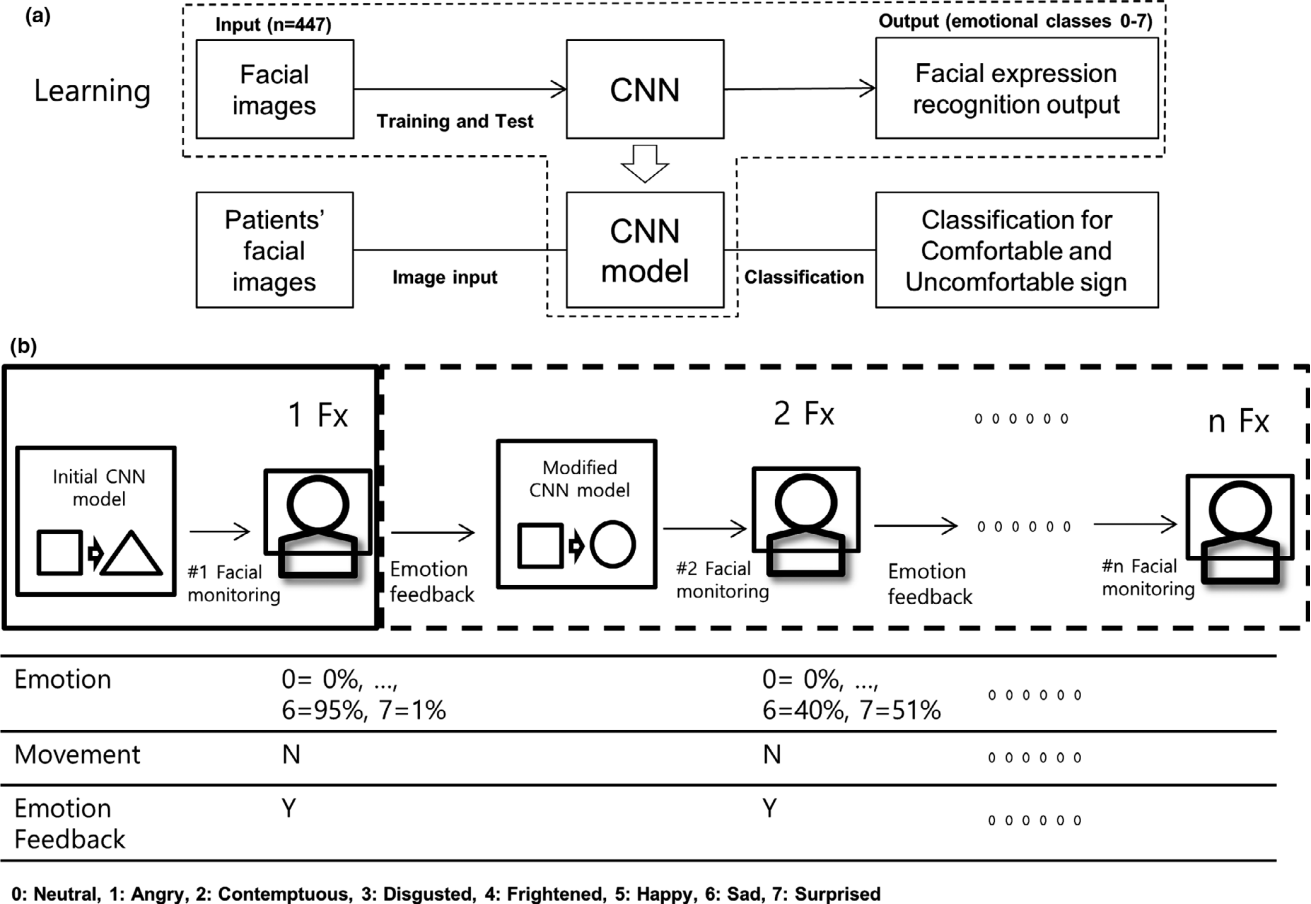
Implemented system algorithm using the convolutional neural network and the entire process for the whole treatment.

To classify the facial expression of the patient, the monitoring system collected 447 source images from CK+ dataset that have already classified and labeled the facial expressions for each photograph of 123 people, and training was performed on the CNN. In order to classify eight expressions, CNN was trained to minimize cross‐entropy. Using the CNN, which finally underwent the training, patients' comfortable and uncomfortable statuses by recognizing their facial images in the treatment room were distinguished [Fig. 4(a)]. Currently, the method used in this study aimed to monitor facial expression only in the first fractional treatment of patients by using the first constructed CNN model. At this time, the percentage of recognition of each facial expression was quantified, and it was whether the actual movement took place was recorded. However, to operate the facial expression monitoring system in a customized manner, it was fed back to the system to be used in the next fraction treatment, enabling the correction for the presence of movement for the patient’s facial expression monitored up to the last treatment time point, and this was repeated until n−1 fraction treatment [Fig. 4(b)]. The feedback was provided by calculating the weight to consider the ratio of uncomfortable dominant emotion and other emotional event sum counted.

### 2.C Training data and test dataset

**Fig. 5:**
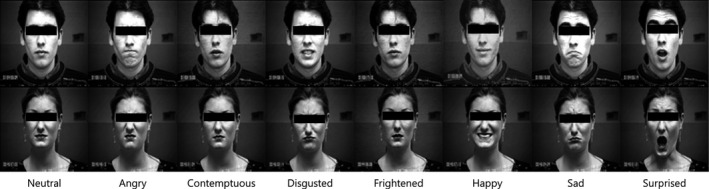
Sample facial images from 447 images (©Jeffrey Cohn). *Notation: Red = dominant emotion for the facial expression score over 50% for each frame.

The training dataset used for the learning of CNN was CK+ dataset.^24^, ^25^ The dataset consists of 447 facial expressions of the 640 × 480 source images and are composed of eight facial expressions: neutral, angry, contemptuous, disgusted, frightened, happy, sad, and surprised. The CK+ dataset is only used for classifying the facial expressions and it does not have any patient movement information. For each image, the corresponding emotion is labeled (Fig. 5). At this time, the emotion composition of the facial images is shown in Table 2.

**Table 2:** Facial expression composition.

Neutral	Angry	Contemptuous	Disgusted	Frightened	Happy	Sad	Surprised	Total
121	45	18	59	25	69	28	82	447
27.07%	10.07%	4.03%	13.20%	5.59%	15.44%	6.26%	18.34%	100%

The CNN training time was 3 ms per 1 epoch and total time was 10 min per 200 epochs. And the computer equipped with Intel® Xeon® E5‐2650 V4 x2 (256 GB RAM, NVIDIA GeForce GTX 1080 Ti x2 GPU) was used. Only 80% of the 447 data from the image sources were used for the training, and the remaining 20% were used to assess the accuracy of the CNN. In order to train the CNN efficiently, it is necessary to reduce the computational amount of the data set. Therefore, the training image was downsized to 48 × 48 size by cropping only the face part. The prediction time was 12 ms and processing time per each frame was 57 ms. The patient's images were analyzed frame by frame and processed in real time using four multi‐threading. Therefore, time lag is negligible.

### 2.D Real test sample with the patients

Ten patients (male = 7, female = 3, average age = 67.3 ± 6.72) were tested for recognition rate using our system in the treatment room. The patients should not wear thermo‐plastic mask, so they were selected for treatment while in head‐first‐supine position, except for head and neck cancer site (liver = 5, esophagus = 5).

## 3 RESULTS

### 3.A Facial expression and movement monitoring results

**Fig. 6:**
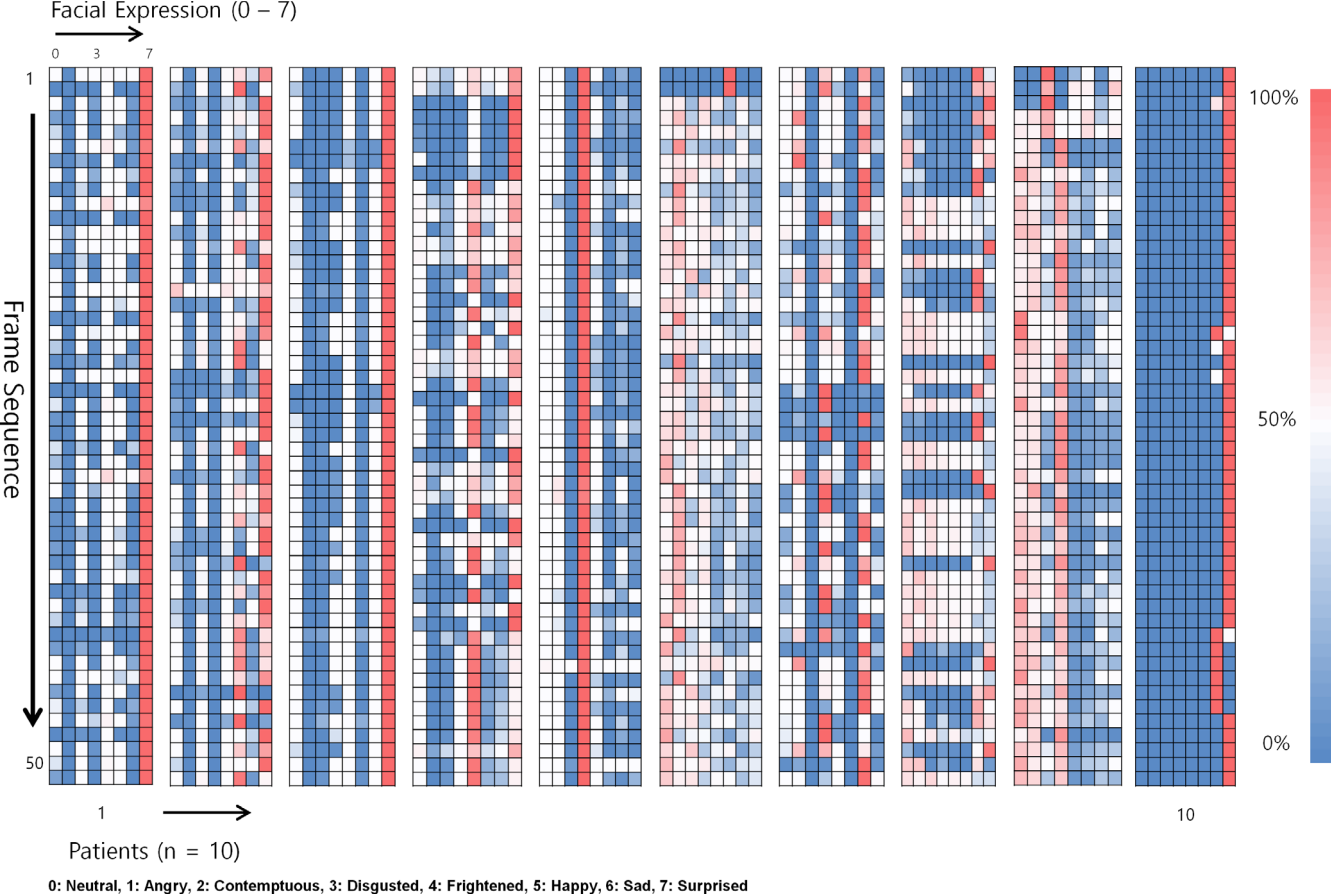
Facial expression monitoring result by frame sequence (n = 10).

The images trained by input in CNN's algorithm model were images with a camera recognition angle facing front. The images used for actual patient monitoring were taken from the forehead direction at a high angle from 30 to 60 degree angle. For the ten recognized patients, the comfortable expression (neutral, frightened, and happy) and uncomfortable expression (sad, contemptuous, angry, surprised, and disgusted) were clearly differentiated. Figure 6 shows that the heatmap represents various emotions for the 10 patients by extracted 50 frame sequences. The red line indicates the dominant emotion for the facial expression score over 50% for each frame for the patients. However, during the 3‐minute monitoring period, the patient’s facial expressions changed from time to time, and few patients were treated with the comfortable expressions we categorized in this study.

In Table 3, it showed that the recorded eight patients were treated with an uncomfortable expression, and no sudden movement of the patient was detected. Stability is the dominant emotion per rest of emotions during monitoring. This means that most patients were treated with various expressions without maintaining a constant expression for a certain time. And the expected nonmotion scores that the comfortable emotions scoring results not to move in all frames for each patient. Thus, the patients who had comfortable and neutral emotion had higher score. And the expected motion is opposite situation of non‐motion. Therefore, motion prediction accuracy follows the expected non‐motion according to the movement result of the patient, since the movement of the patient did not appear (Table 3).

**Table 3:** Facial recognition results.

n	Detection	Dominant emotion (%)	Stability (%)	Expected nonmotion (%)	Expected motion (%)	Real motion result	Motion prediction accuracy (%)
1	Y	Sad (53)	88.6	1.0	99.0	N	1.0
2	Y	Surprised (67.2)	48.9	0.2	99.8	N	0.2
3	Y	Happy (54.1)	84.8	92.7	7.3	N	92.7
4	Y	Disgusted (77.4)	29.2	5.8	94.2	N	5.8
5	Y	Sad (63.6)	57.3	5.4	94.6	N	5.4
6	Y	Disgusted (86.1)	16.1	11.1	89.0	N	11.1
7	Y	Surprised (55.3)	80.8	27.7	72.3	N	27.7
8	Y	Disgusted (72.6)	37.8	2.8	97.2	N	2.8
9	Y	Surprised (72.6)	37.8	24.6	75.4	N	24.6
10	Y	Neutral (55.7)	79.7	75.5	24.5	N	75.5

^a^ Notation: Stability = Dominant emotion/(1 − dominant emotion).

### 3.B Accuracy test

**Fig. 7:**
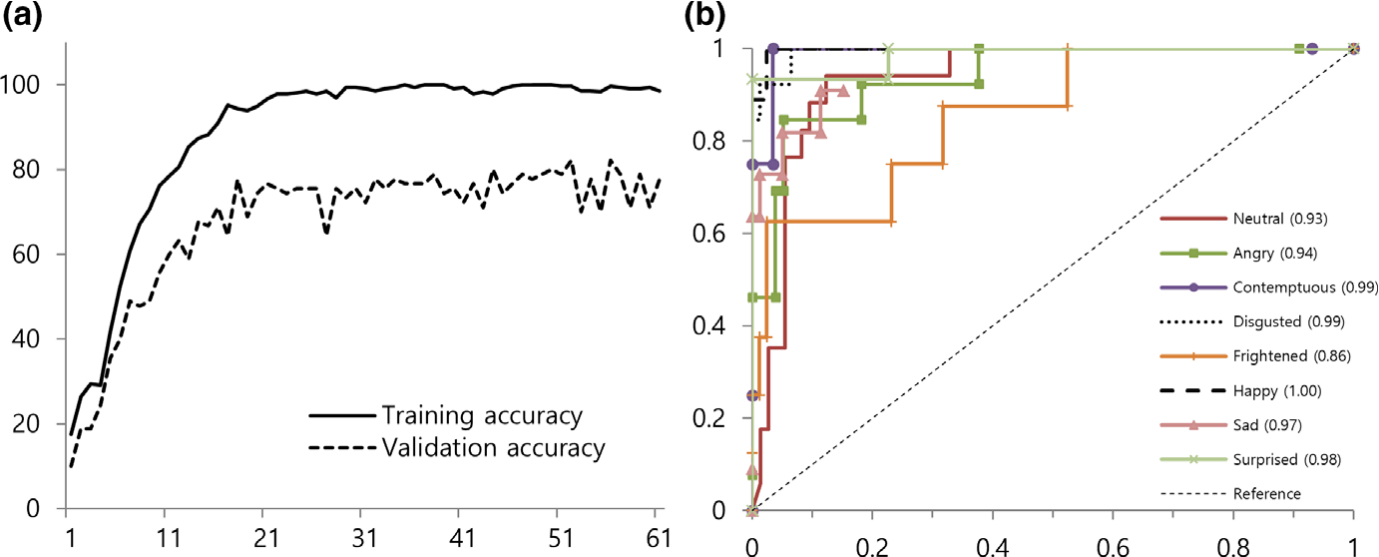
Training, validation accuracy, and receiver operating characteristic curve for facial emotions.

To evaluate the accuracy of the system, we analyzed the training and validation accuracy of the CNN model. Moreover, the receiver operating characteristic curve analysis was performed for each facial expression group. To evaluate the performance of the system, the recognition rate was tested using a camera with a developed CNN model. Subsequently, monitoring was performed on the patients in the actual treatment room to analyze the recognition rate. The results of the accuracy analysis of the developed CNN model are shown in Fig. 7. Training accuracy was 100% at maximum, and validation accuracy was 82.2% [Fig. 7(a)]. Test accuracy was 85.6%. The ROC curves for each motion identified by output are shown in Fig. 7(b), with an average of 95.8%, which is a measure of how accurately each emotion is identified in the model.

## 4 DISCUSSION

The initial CNN model defined and categorized comfortable facial expressions (neutral, frightened, and happy) and uncomfortable facial expressions (sad, contemptuous, angry, surprised, and disgusted) and classified them accordingly. However, in 10 recognized patients, only 20% of patients were treated with comfortable facial expressions in the initial CNN model. A total of 60% of patients were treated with an uncomfortable facial expression, and the remaining 20% showed various emotions on their face. In this case, since the sudden movement of the patient was not detected, the system defined in the first modeling needs modification of uncomfortable expressions to comfortable expressions as the treatment fraction number increases according to the facial expression status for each patient. Otherwise, a warning message will be given to indicate that even if the number of fraction treatments increased, the patient still feels uncomfortable. Additionally, statistical analysis and the following correlation studies are required to identify which emotional state the patient showed actual movements. In the monitoring scheme shown in Fig. 4(b), as the treatment fraction progresses, the patient's facial expression results from the previous treatment will be fed back to the monitoring system at the next treatment, resulting in a more complete patient‐specific system as the number of fraction treatments increases.

The recognition rate of the patient's facial expression depends on the image angle. The angle of the image used in the source was obtained to allow an angle of 30° in the front direction, and it was not recognized if it was out of the screen angle, higher than 60° of the image. Therefore, we selected an angle so that the eyes, nose, and mouth were captured by the camera in the forehead direction. If training is performed using a dataset with free‐screen angle, the recognition rate of the system is expected to be improved, and further study on this matter is required.

When the current system is applied for clinical applications, it is limited in patients undergoing head and neck stereotactic radiosurgery who wear a head thermoplastic frame or a stereotactic head frame and in patients assuming a prone position. And only ten patients were tested in the treatment room while they are treating as a preliminary study. In further study, more patient cases will be analyzed after final installation in the treatment room.

In a commercial system capable of surface imaging, it is possible to monitor patient’s motion in real time. However, in the treatment environment where surface imaging is difficult, the system developed and proposed in this study can be implemented efficiently.

The patient’s face recognition can be used in the process of identifying the patient in conjunction with the image taken at the time of the patient’s visit. It could be implemented as part of an intelligent patient treatment system by combining it into the total information process in the treatment stage such as treatment setup.

## 5 CONCLUSIONS

We have developed a system that recognizes patient’s facial expressions. The system detects the uncomfortable emotion of a patient using AI algorithms and gives advance warning to the radiation treatment technologist on the possibility of movement of the patient as a result of the uncomfortable condition of the patient. If the recognition rate and accuracy of the system are improved and further studies are conducted, we confirmed that the system could be actively used in the treatment of patients.

## CONFLICT OF INTEREST

The authors declare no conflict of interest.
